# A Randomized Controlled Trial for the Effectiveness of Progressive Muscle Relaxation and Guided Imagery as Anxiety Reducing Interventions in Breast and Prostate Cancer Patients Undergoing Chemotherapy

**DOI:** 10.1155/2015/270876

**Published:** 2015-08-06

**Authors:** Andreas Charalambous, Margarita Giannakopoulou, Evangelos Bozas, Lefkios Paikousis

**Affiliations:** ^1^Cyprus University of Technology, 15 Vragadinou Street, 3041 Limassol, Cyprus; ^2^University of Athens, 123 Papadiamantopoulou Street, 115 27 Athens, Greece; ^3^Improvast, 7 Arkadias Street, Office 206, 2020 Nicosia, Cyprus

## Abstract

*Objective*. To test the effectiveness of guided imagery (GI) and progressive muscle relaxation (PMR) as stress reducing interventions in patients with prostate and breast cancer who undergo chemotherapy. *Methods*. Patients were randomly assigned to either the control group or the intervention group (PMR and GI). Patients were observed for a total duration of 3 weeks and assessed with the SAS and BECK-II questionnaires for anxiety and depression, respectively, in addiotion to two biological markers (saliva cortisol and saliva amylase) (trial registration number: NCT01275872). *Results*. 256 patients were registered and 236 were randomly assigned. In total 104 were randomised to the control group and 104 to the intervention group. Intervention's mean anxiety score and depression score changes were significantly different compared to the control's (*b* = −29.4, *p* < 0.001; *b* = −29.4, *p* < 0.001, resp.). Intervention group's cortisol levels before the intervention (0.30 ± 0.25) gradually decreased up to week 3 (0.16 ± 0.18), whilst the control group's cortisol levels before the intervention (0.21 ± 0.22) gradually increased up to week 3 (0.44 ± 0.35). The same interaction appears for the Amylase levels (*p* < 0.001). *Conclusions*. The findings showed that patients with prostate and breast cancer undergoing chemotherapy treatment can benefit from PMR and GI sessions to reduce their anxiety and depression.

## 1. Introduction

Cancer itself and its treatments have been evidently considered as sources of anxiety and depression for patients [1, 2]. In a recent study Salvo et al. [3] found that 55% of cancer patients reported at least mild levels of depression and 64% reported at least mild levels of anxiety. Depressed and anxious individuals have lower social functioning, more disability, and greater overall functional impairment [4]. Distressed emotional states often generate additional somatic problems including sleep difficulties, fatigue, and pain. Depression and anxiety can even induce behaviour changes and they can affect treatment by impairing cognition, weakening motivation, and decreasing coping abilities and quality of life [4].

The body's stress system includes the catecholamines, norepinephrine, and epinephrine, which are regulated by the sympathetic nervous system (SNS), and the glucocorticoids, which are regulated by the hypothalamic-pituitary-adrenal (HPA) axis. The HPA axis plays an important role in maintaining body homeostasis in response to stress [5]. Stress leads to activation of the HPA axis, increasing peripheral cortisol that is known also to result in depressed mood [6]. The role of salivary cortisol (the most potent human glucocorticoid) as a reliable biological stress marker is recognized for several decades now [7]. The salivary enzyme alpha-amylase (sAA), which is mainly involved in the digestion of starch in the oral cavity, has received increasing attention as a stress marker of the SNS within the last decade [8]. Although sAA is not a direct by-product of the SNS, there exist several studies showing the involvement of the autonomic nervous system (ANS), particularly its sympathetic branch, in the sAA secretion process [5].

In an effort to cope with the sources of anxiety and depression patients often seek medication [9], psychotherapy [10], and cognitive behavioural techniques that fall under the category of Complementary and Alternative Medicine (CAM) that in the recent years has become a popular trend especially among patients [11]. This trend is in line with current thinking based on the mind-body connection as well as cognitive behavioural techniques utilised in many therapeutic settings. Preceding studies have shown that various psychological interventions can help the patient gain a better sense of control over distressing symptoms and side-effects of cancer. Such interventions include basic cognitive restructuring, hypnotherapy, art and music therapy, progressive muscle relaxation (PMR), and guided imagery (GI) just to report a few. For most of these interventions including PMR and GI researchers have produced mixed evidence for their effectiveness in managing anxiety and depression, an aspect that limited their utilisation in clinical practice. Adding to the discussion the underutilisation of PMR and GI can also be attributed to the methodological flaws often identified in these studies that impact the quality of the findings [12, 13]. These include the use of heterogenous patients' populations, small sample sizes, nonrandomised samples, poor quality of the intervention design and application, sole use of psychometric assessment, short period of implementation, lack of control over extraneous factors, poor patients' adherence to the intervention, and poor randomisation process.

In a community-based nursing study in Australia, Slomar [14] compared the effects of PMR and GI, depression, and quality of life in people with advanced cancer. Fifty-six people with advanced cancer who were experiencing anxiety and depression were randomly assigned to 1 of 4 treatment conditions: (a) progressive muscle relaxation training, (b) guided imagery training, (c) both of these treatments, and (d) control group. Subjects were tested before and after learning PMR and GI techniques for anxiety, depression, and quality of life. No significant improvement for anxiety was found; however, significant positive changes occurred for depression and quality of life. Campbell-Gillies [15] used a program including positive mental images and music with 45 women with breast cancer. Her findings revealed that GI decreased depression and anxiety over a six-cycle period of chemotherapy. Loizzo et al. [16] explored the effectiveness of a 20-week meditation-focused intervention in reducing distress and disability in 46 breast and gynaecologic cancer patients who had completed chemotherapy. Social support, anxiety, depression, adjustment to cancer, and quality of life were all examined before and after test. Biological measures including cortisol levels, resting heart rate, natural killer cell, and interleukin-6 levels were also examined before and after test. Results demonstrated improvement in anxiety, depression, and quality of life scores.

The aim of this study was to provide evidence on the effectiveness of a PMR and GI program in patients with breast and prostate cancer undergoing chemotherapy. This study was designed and implemented with the purpose of providing both psychometric and biological (saliva a-amylase and saliva cortisol biomarkers) evidence on the effectiveness of the program in breast and prostate cancer groups.

## 2. Methods

### 2.1. Study Design, Participants, and Settings

This was a randomised controlled trial with prospective participants recruited from three cancer centres in Cyprus being assigned either to the intervention or the control group. Patients were eligible to participate if they had a histopathology diagnosis of breast (clinical stage T3N1M0) or prostate cancer (clinical stage T3a, Gleason score ≥ 8), were experiencing anxiety and depression (referred to the study by centres' psychiatrist), were over 18 years of age, were not receiving medication for anxiety or depression, were willing to participate, were able to speak and write Greek, did not have a cognitive impairment, did not have hearing or sight problems, and were receiving chemotherapy. Patients were excluded if they had xerostomia or/and oral mucositis as these two conditions interfere with the sAA and saliva cortisol levels. Patients receiving high doses of cortisone were also excluded by the study. Patients that during the study required medication for anxiety or depression were excluded. The trial was overseen by a trial management group and reviewed by an independent data-monitoring committee every 12 months. At each review the data-monitoring committee and the Bioethics Committee recommended that the trial could continue. No formal stopping rules were used. This study was done in compliance with the Declaration of Helsinki [17] and the protocol was approved by the Cyprus National Bioethics Committee (CNBC/EP/2010/06).

### 2.2. Randomisation and Masking

Consenting patients were randomly assigned in a 1 : 1 ratio to experimental or control arm using a computer-based minimisation algorithm stratifying for cancer type (prostate or breast) and the cancer centres. Participants were not masked to the allocated treatment because blinding was not practicable. The assessors were blinded, so at the moment of the various assessments they were unaware of which group the patients belonged to.

### 2.3. Intervention and Procedures

**Figure 1:**
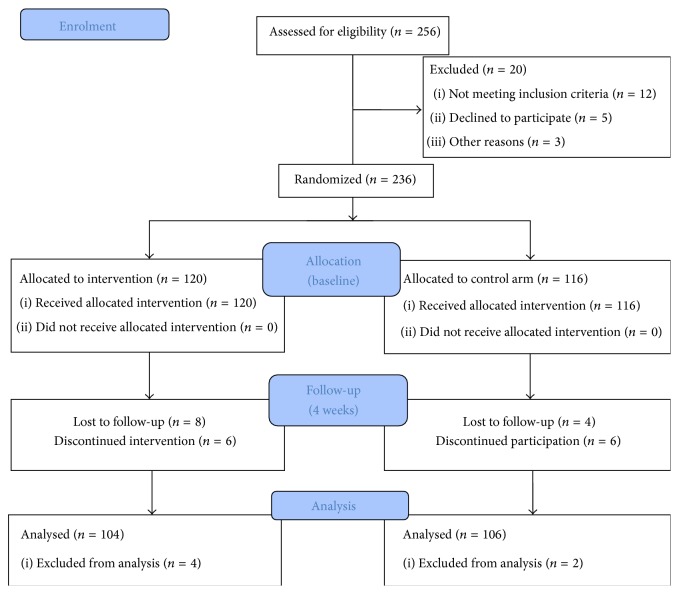
Trial profile. CONSORT flow diagram.

The intervention entailed a combination of PMR and GI sessions both supervised and unsupervised. Supervised sessions were carried out by experienced research assistants with prior training in these techniques. The intervention sessions took place at the participants' homes as to minimise any extraneous stressful factors generated by the medical environment of the hospital. All patients gave written informed consent. Following randomization, participants in the control group received standardised care (weekly meetings with centre's psychologist) and participants in the intervention group received 4 supervised sessions of PMR and GI in addition to daily unsupervised sessions for a duration of three weeks (Figure 1: trial profile). Daily reminders (text messages) were given to patients in the intervention group to remind them of practicing PMR and GI every day at the same time.

In the context of this study, GI is defined as a cognitive process that utilises the imagination to bring about positive mind/body responses that stimulate the senses [18]. PMR is defined as a technique of alternately tensing and relaxing muscle groups in sequence throughout the body to induce relaxation, a state of freedom from anxiety and skeleton muscle tension [19].

As part of this study the researchers developed and tested a new GI scenario which could address a major limitation previously identified by researchers, the fact that a substantial number of people are unable to visualize images [20]. Therefore in order to stimulate the visualisation process the developed intervention included auditory, tactile, and olfactory images. The GI script described the smooth ascending and descending of the patient on the sky and the viewing of the scenery from above. The scenario was dressed with music which camouflaged alpha waves pulses that bring the mind to a relaxed but at the same time conscious state [21]. As it was acknowledged that some participants might have had a fear of height an alternative script was developed that described the scenery of a peaceful beach. A PMR scenario was also developed involving the exercise of 11 group muscles progressively moving from the feet to the muscles located on the face. The intervention was tested prior to the study in two groups, a group of 15 nursing students and a group of 9 volunteer cancer patients. Their opinions were recorded especially as to the easiness to follow the images projected and the ability of the intervention to produce relaxation. Their response to the test was also measured with Biodots [22] that provide biofeedback (respond to skin temperature changes). After the intervention the participants were asked to describe their experiences and based on these alternations were made to the GI script whilst no changes were indicated for the PMR or the breathing exercises. Once changes were integrated, a final test to the same groups followed. No further changes were indicated. The final version of the intervention included a 2-minute breathing exercise, followed by a 10-minute PMR exercise and a 15-minute pleasant GI session.

### 2.4. Outcomes

Both groups were assessed at baseline and at the end of the three-week intervention period for their anxiety and depression levels with Greek validated scales [23, 24]. Anxiety as the primary outcome was assessed by the Zung Self-Rating Anxiety Scale (SAS) [25] and depression was measured by the Beck Depression Interview II (BDI-II) [26]. The Zung SAS is a 20-item, self-report questionnaire that measures the presence and magnitude of anxiety-based symptoms.

The SAS was constructed according to the* DSM-II* criteria for anxiety and still contains the criteria listed in* DSM-IV-TR* (APA, 2000), giving it good content and face validity [27]. It contains items that assess both physiological (e.g., muscle tremors, physical pain, and urinary frequency) and psychological (e.g., nervousness, fear, and mental disintegration) symptoms commonly associated with anxiety [25]. Each item is scored on a 4-point scale in relation to whether the person has experienced each specific symptom* none or a little of the time* (rating = 1),* some of the time* (2),* a good part of the time* (3), or* most or all of the time* (4) during the last two weeks. There are positively and negatively worded items to reduce response bias and identify inconsistencies in responses. Raw scores sum to 20–44 denoting normal anxiety levels, 45–59 denoting mild to moderate anxiety levels, 60–74 denoting marked to severe anxiety levels, and 75–80 indicating extreme anxiety levels.

The BDI-II consists of 21 items each designed to assess a specific symptom common among people with depression [26]. The inventory was primarily developed to measure the intensity, severity, and depth of depression in patients. The questions come with four possible responses ranging from zero to three, indicating the severity of the symptom. The responses are then summed to indicate the severity of depression with the interpretation being as follows: 1–10 is considered normal, 11–16 indicated mild mood disturbance, 17–20 indicates borderline clinical depression, 21–30 represents moderate depression, 31–40 represents severe depression, and over 40 indicates extreme depression.

*Collection of Salivary Samples and a-Amylase and Cortisol Measurements*. Whole saliva samples were collected with the Salimetrics Oral Swab, P/N 5001.02 (SOS Salimetrics Ltd.) indicated for the biomarkers tested in this study. A baseline measurement was taken from the participants in both groups and three weekly assessments followed. It is evident in the literature that humans exhibit daily physiological and behavioural rhythms with nearly all body functions including the secretion of cortisol and sAA [27, 28]. In order to address the potential influence of these circadian rhythms, saliva samples were taken from each participant at the same time of the day throughout the study. The participants were instructed to place the oral swab under the tongue for at least 2 minutes [29]. Once removed, the swabs were placed in tubes and frozen at −20°C within one hour of collection. On the day of testing, the samples were brought to room temperature and then centrifuged for 15 minutes at 1500 g.

Cortisol (ng/mL) levels were quantified by commercially available competitive immunoassay kit (Salimetrics, State College, PA, catalog number 1-3002). a-Amylase (ng/mL) levels were measured by commercially available kinetic reaction assay kit (Salimetrics, State College, PA, catalog number 1-902-5). All assays were run in duplicate. Intra- and interassay coefficients of variation were less than 5% and 10%, respectively.

### 2.5. Statistical Analysis

Sample size was indicated by power analysis and equal numbers of patients were assigned to the two groups. The planned sample size (100 patients in each group) was calculated to detect a difference of 10 points in the anxiety scale in the intervention group, in the hypothesis of mean 45 points in the control group, and a common standard deviation of around 23 points, with power 80% and alpha error 5% by a Student's *t*-test for independent samples. We used a two-sided 5% significance level for all analyses.

Comparison between control and intervention groups at baseline and weekly measurements for sAA and cortisol levels were examined using a *t*-test for independent samples. Effect size differences on weekly measurements are reported using the Cohen's *D* statistic. To quantify the magnitude of the effect on the two biomarkers, effect size difference between intervention and control was calculated at each weekly measurement. The interaction between treatment group and weekly measurements of the biomarkers was assessed with Generalised Estimating Equations analysis using an unstructured correlation matrix [30]. Variations of the correlation structure produced similar results but with slightly poorer model fit based on the QIC criterion.

For a given scale (SAS or BDI-II), the individual difference between baseline and the end of intervention (3rd week) was calculated. Generalised Estimating Equations analysis was also used, to assess whether the change (development from baseline to the 3rd week measurement) in anxiety and depression was different across the 2 groups.

## 3. Results

### 3.1. Demographics and Clinical Characteristics

The two groups were generally well-matched at baseline for key demographic variables. Between January 2011 and December 2012, 256 patients were assessed for eligibility and 208 patients successfully completed all the assessments in the study. The sample consisted of 104 male (52 in the intervention group and 52 in the control group) and 104 female (52 in the intervention group and 52 in the control group) patients diagnosed with prostate and breast cancer, respectively (Figure 1: trial profile). The majority of the prostate cancer patients (83%) were diagnosed with stage T3a, Gleason score 8, and the remaining 17% with stage T3b, Gleason score 9. Patients with breast cancer were all diagnosed with clinical stage T3N1M0. Seventy-one percent of the participants were married (151) and 87% reporting that their spouse and children were their primary caregivers. Participants came from all the geographical regions in Cyprus with most participants coming from Nicosia (53.8%) and the least from Ammochostos (28.2%). Seventy-three participants belonged to the 51–60 years age group (68.8%) followed by the 40–50 age group (62.3%). Fifty-two participants (49%) had university education and 18 (18.9%) had no education.

### 3.2. Internal Consistency Reliability

Internal consistency of the SAS and Beck-II scales was tested with the Cronbach's alpha. Anxiety scale before the intervention (*a* = 0.85) and after the intervention (*a* = 0.95) demonstrated an excellent consistency. Depression scale before the intervention (*a* = 0.77) and after the intervention (*a* = 0.94) also demonstrated an acceptable and excellent consistency, respectively.

### 3.3. Primary Outcome

The intervention group demonstrated a decrease in its mean anxiety score from baseline (45.01 ± 6.9) to 3 weeks after the intervention (38.71 ± 6.1) while the control group demonstrated an increase in its mean anxiety score from baseline (39.47 ± 9.9) to 3 weeks after the intervention (44.38 ± 7.6) (Table 2). Intervention's mean anxiety score change was significantly different compared to the control's (*b* = −29.4  *p* < 0.001) (Table 1).

Based on the distribution of patients with regard to the anxiety categories (normal, moderate, severe, and extreme), in the intervention group, the number of people in the normal range was the majority numbering 86 participants. The number of people in the mild to moderate anxiety levels has decreased from 56 (before intervention) to 18 (after the intervention). No people with severe anxiety levels were recorded after the intervention in this group. Contrary to the control group, the people in the mild to moderate anxiety levels increased from 32 to 49 as well as the people reporting severe anxiety levels (from 2 to 12).

### 3.4. Secondary Outcome

The intervention group demonstrated a decrease in its mean depression score from baseline (39.07 ± 9.9) to 3 weeks after the intervention (21.35 ± 8.5) while the control group demonstrated an increase in its mean depression score from baseline (25.39 ± 17.5) to 3 weeks after the intervention (37.10 ± 12.3) (Table 2). Intervention's mean depression score change was significantly different compared to the control's (*b* = −29.4  *p* < 0.001) (Table 1).

Based on the distribution of patients with regard to the depression categories (minimal, mild, moderate, and severe), for the intervention group, only 1 person had minimal depression and after the intervention the number increased to 12. Severe depression was observed in 89 persons while after the intervention the number dropped to 20. Contrary to the intervention group, in the control group the people reporting moderate (from 16 to 26) and severe depression (from 47 to 80) increased in the period following the intervention.

### 3.5. Biomarkers

**Figure 2:**
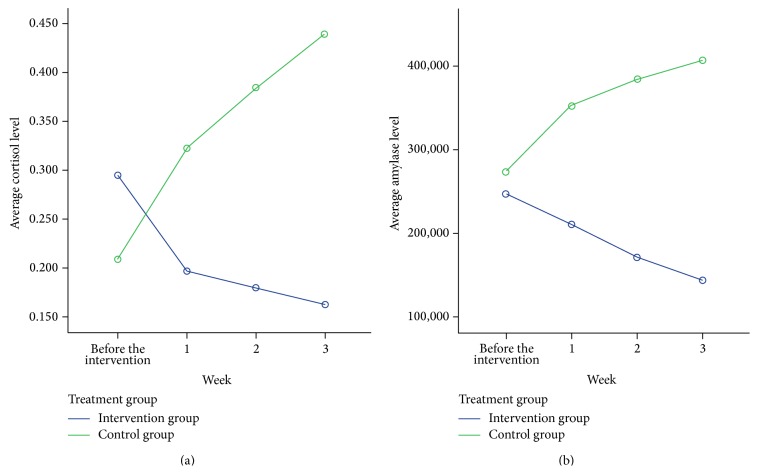
Average level of biomarkers in baseline and in weekly measurements by treatment group. The interaction effect is obvious for both biomarkers and statistically verified using the GEE analysis (*p* < 0.001). Cortisol and amylase levels are increasing for the control group and decreasing for the intervention group.

There is an interaction between treatment group (intervention/control) and the weekly average cortisol level (*p* < 0.001) (Table 3) (Figure 2). Specifically, the intervention group's cortisol levels before the intervention (0.30 ± 0.25) gradually decreased up to week 3 (0.16 ± 0.18). On the other hand, the control group's cortisol levels before the intervention (0.21 ± 0.22) gradually increase up to week 3 (0.44 ± 0.35) (Table 2).

The same interaction appears for the amylase level (*p* < 0.001) (Table 3). The intervention group's sAA levels before the intervention (246.9 ± 145.7) gradually decrease up to week 3 (142.7 ± 99.5). The sAA levels before the intervention for the control group (237.5 ± 146.1) gradually increase up to week 3 (407.3 ± 184.6) (Table 2).

In terms of the magnitude on the two biomarkers, at week 3, the effect size between intervention and control for the cortisol level was Cohen's *d* = 0.99 indicating a strong difference. Effect on amylase levels, on the other hand, is nearly double (Cohen's *D* = 1.79).

### 3.6. Scale and Biomarker Association

Before the intervention, anxiety scale correlates with depression scale (*r* = 0.62  *p* < 0.001) as well as with cortisol (*r* = 0.32  *p* < 0.001) or amylase (*r* = 0.41  *p* < 0.001). Depression scale also correlates with cortisol (*r* = 0.28  *p* < 0.001) and amylase levels (*r* = 0.35  *p* < 0.001). Amylase and cortisol correlate (*r* = 0.29  *p* < 0.001).

At the end of 3rd week, after the intervention, anxiety scale correlates with depression scale (*r* = 0.59  *p* < 0.001), cortisol at 3rd week (*r* = 0.22  *p* < 0.001), and amylase at 3rd week (*r* = 0.29  *p* < 0.001). Depression scale also correlates with cortisol at 3rd week (*r* = 0.39  *p* < 0.001) and amylase at 3rd week (*r* = 0.34  *p* < 0.001). Cortisol and amylase at 3rd week also correlate (*r* = 0.52  *p* < 0.001).

## 4. Discussion

In the literature anxiety and depression have been frequently associated with patients receiving chemotherapy [31]. Anxiety and depression can compromise compliance with treatment and negatively affect overall quality of life, prognosis, and survival rates [32]. Cultural or societal factors pose an additional challenge to the recognition and reporting of anxiety and depression by cancer patients increasing the problem of underrecognition [33, 34]. Furthermore, the reluctance on behalf of the patients to seek treatment for anxiety and depression also contributes to the overall problem [35].

The problem of anxiety and depression in this group of patients calls for a more comprehensive management that extends beyond the use of solely pharmacological interventions. Various nonpharmacological measures have been tested as adjuvant treatments for reducing anxiety and depression, including massage [36], Reiki [37], and cognitive behavioural interventions such as PMR and GI [14]. Progressive muscle relaxation and GI techniques either separately or in combination have been studied in many different modalities and cancer groups for their effectiveness in strengthening the immune system [38], improving quality of life [39], and controlling various cancer and treatment related side-effects such as pain [40], fatigue [41], and nausea and vomiting [42, 43]. A few studies also explored PMR and GI benefit in managing anxiety and depression. Linde and Stuart [44] created a study that examined how cognitive-relaxation-visualization affected anxiety in women with breast cancer. They looked at two different groups: women who were before diagnosis with breast lumps and were undergoing mammograms, and women who were after diagnosis who had surgery and were about to undergo radiation therapy. Results showed that the intervention reduced anxiety in both pre- and postdiagnosis breast cancer patients. Patients felt that the intervention provided a distraction from their current situation and helped relieve anxiety [44].

León-Pizarro et al. [45] in a randomised trial with 66 patients programmed to receive brachytherapy explored the effectiveness of training in relaxation and guided imagery on anxiety and depression. The intervention group demonstrated a statistically significant reduction in anxiety (*p* = 0.008), depression (*p* = 0.03), and body discomfort (*p* = 0.04) compared with the control group. The benefits of PMR and GI were also found in other patient groups. Mizrahi et al. [46] studied the effect of PMR and GI on anxiety and quality of life among patients with inflammatory bowel disease. In a prospective, randomised control trial, 56 outpatients were randomly chosen and allocated to a treatment group or a waiting-list control group. Treatment group patients attended three relaxation-training sessions and received an audio disc for home practice. Following the intervention, the treatment group's (*n* = 18) measured results showed a statistically significant improvement as compared to the control group (*n* = 21): anxiety levels decreased (*p* < 0.01) and QoL and mood improved (*p* < 0.05), while levels of pain and stress decreased (*p* < 0.01). Apóstolo and Kolcaba [47] tested the efficacy of a GI intervention for decreasing depression, anxiety, and stress and increasing comfort in psychiatric inpatients with depressive disorders. A quasi-experimental design sampled 60 short-term hospitalized depressive patients selected consecutively. The experimental group listened to a guided imagery compact disc once a day for 10 days. The Psychiatric Inpatients Comfort Scale and the Depression, Anxiety, and Stress Scales (DASS-21) were self-administered at two time points: prior to the intervention (T1) and 10 days later (T2). Comfort and DASS-21 were also assessed in the usual care group at T1 and T2. Repeated measures revealed that the treatment group had significantly improved comfort and decreased depression, anxiety, and stress over time. Overall the relevant literature shows that PMR and GI have been used as stress reducing interventions. A reason for this lays in their simplicity and the ease to implement them without supervision. However, the fact that people vary in terms of their ability to visualize images is a reason that can affect the effectiveness of GI and one that needs consideration by researchers.

To the best of our knowledge, this is the first randomised controlled clinical trial to simultaneously assess the effectiveness of a combined PMR and GI program in managing anxiety and depression in patients with prostate and breast cancer receiving chemotherapeutic agents. Patients in the intervention group performed a combination of relaxation and visualisation exercises once a day for 3 weeks. They were evaluated (psychometric and biological) immediately before they began chemotherapy, when they completed 7 intervention sessions (biological only) and 14 intervention sessions (biological only), and when they completed 21 sessions (psychometric and biological). The study has incorporated the use of biomarkers as a means to assess the patients' body response to the intervention. The intervention protocol was developed via a rigorous process in order to meet the explicit needs reported by this group of patients. Other strengths of the study included the trial design (randomised), adequate statistical power, comparable study groups, and longitudinal assessment of patients. These strengths allow for replication of the study and generalizing the results to the same group of patients that go through chemotherapy and experience various levels of anxiety and depression.

The findings of this study add to the relevant literature [48, 49] of the use of salivary cortisol and sAA as meaningful and reliable stress markers indicative of HPA axis function and SNS function, respectively. This study also provided evidence that support the association of salivary cortisol and sAA, indicating that the HPA axis and SNS work in coordination to mediate the physiologic response to a perceived stressor [30]. This association has not been consistently found due to salivary cortisol and sAA distinct temporal dynamics [5]. Correlations between salivary cortisol and sAA were found in this study both at baseline and at 3rd week.

The study should be read in light of some limitations. The participants were not blinded as part of this trial and only the assessors were blinded. This study design is inferior to double blind randomized controlled trial as it is difficult to control for placebo effect. However, the comparability of baseline characteristics for both intervention and control groups assured the researchers that the observed improvement was due to the intervention itself. Patients in the intervention group received daily text messages as a reminder to practice PMR and GI every day at the same time. Furthermore, patients were encouraged to keep a personal diary as part of this study. By reviewing the comments recorded by the patients in their diaries it became apparent that the intervention was well accepted and utilised consistently by the patients. However, the researchers are not sure if these activities influenced participant anxiety and depression. Furthermore, it is uncertain if the fact that the patients in the intervention group were required to perform something on a daily basis and repeatedly resulted in the differences found in this study or if these could be attributed to the relaxation and visualization exercises.

In conclusion, patients diagnosed with cancer and receiving chemotherapy experience anxiety and depression especially when the disease has progressed and the prognosis is poor. Despite the high prevalence of anxiety and depression in these patients groups there have been few intervention strategies considered.

Although the mechanisms by which cognitive behavioural interventions can modify or interfere with the stress response to external stimuli still remain unknown, PMR in combination with GI is more effective than standard treatment alone in patients diagnosed with breast and prostate cancer receiving chemotherapy. Therefore, the researchers recommend that healthcare professionals implement or recommend to patients undergoing chemotherapy these techniques as an adjuvant mean to minimise their anxiety and depression. Cancer patients continue to experience elevated levels of emotional distress even after cancer treatment. Taking this into consideration in addition to the fact that the intervention's effect increased over time it is essential for the intervention to be continued after the hospital discharge, although the long lasting effects of the PMR and GI were not tested in this study. In order to guarantee this continuous implementation it is important to improve the community network of cognitive behavioural care facilities and to educate health care professionals in cognitive behavioural skills.

## Figures and Tables

**Table 1:** Generalised Estimating Equations parameter estimates for the effect of intervention on depression and anxiety. The group by time interaction is verified for both scales. There is difference in the linear effect of time between intervention and control for the depression scale (*b* = −29.423) and for the anxiety level (*b* = −11.212).

Parameter	*B*	SE	Wald chi-square	*p* value
Dependent variable: depression				
(Intercept)	13.692	2.4018	32.498	<0.001
Intervention	43.096	2.8172	234.011	<0.001
Time	11.702	0.8333	197.222	<0.001
Intervention *∗* time	−29.423	1.0952	721.794	<0.001

Dependent variable: anxiety				
(Intercept)	34.558	1.4154	596.135	<0.001
Intervention	16.75	1.8077	85.854	<0.001
Time	4.913	0.5757	72.853	<0.001
Intervention *∗* time	−11.212	0.83	182.442	<0.001

**Table 2:** Average values for anxiety and depression scores before and after the intervention. Average values of biomarkers (cortisol and amylase) across weekly measurements.

	Intervention (*n* = 104)	Control (*n* = 104)	*p* value	Mean difference	Cohen's *D*
	Mean ± SD	Mean ± SD			
Scales					
Anxiety score (Zung), before the intervention	45.01 ± 6.9	39.47 ± 9.9	<0.0001	5.54	0.65
Anxiety score (Zung), after the intervention	38.71 ± 6.1	44.38 ± 7.6	<0.0001	−5.67	0.83
Depression score (Beck), before intervention	39.07 ± 9.9	25.39 ± 17.5	<0.0001	13.67	0.96
Depression score (Beck), after the intervention	21.35 ± 8.5	37.10 ± 12.3	<0.0001	−15.75	1.49

Biomarkers					
Cortisol level before intervention	0.30 ± 0.25	0.21 ± 0.22	0.0080	0.087	0.37
Cortisol level at 1st week	0.20 ± 0.18	0.32 ± 0.29	0.0002	−0.127	0.53
Cortisol level at 2nd week	0.18 ± 0.19	0.38 ± 0.31	<0.0001	−0.204	0.79
Cortisol level at 3rd week	0.16 ± 0.18	0.44 ± 0.35	<0.0001	−0.277	0.99
					
Amylase level before intervention	246.9 ± 145.7	237.5 ± 146.1	0.1910	−26.57	0.18
Amylase level at 1st week	210.7 ± 135.8	352.5 ± 165.7	<0.0001	−141.81	0.94
Amylase level at 2nd week	171.2 ± 113.3	383.9 ± 179.8	<0.0001	−212.69	1.42
Amylase level at 3rd week	142.7 ± 99.5	407.3 ± 184.6	<0.0001	−264.67	1.79

**Table 3:** Generalised Estimating Equations parameter estimates for the effect of intervention on cortisol and amylase. The group by time interaction is verified for both biomarkers. There is difference in the linear effect of time between intervention and control for the amylase level (*b* = −72.166) and for the cortisol level (*b* = −0.071).

Parameter	*B*	SE	Wald chi-square	*p* value
Dependent variable: amylase level				
(Intercept)	244.722	14.9182	269.101	<0.001
Intervention	42.749	23.6104	3.278	0.07
Time	37.367	3.3245	126.34	<0.001
Intervention *∗* time	−72.166	5.1623	195.428	<0.001

Dependent variable: cortisol level				
(Intercept)	0.185	0.027	47.117	<0.001
Intervention	0.084	0.0358	5.467	0.019
Time	0.055	0.0094	34.167	<0.001
Intervention *∗* time	−0.071	0.0103	46.644	<0.001
